# A Novel Application of the Hydrophobic Polyurethane Foam: Expansive Soil Stabilization

**DOI:** 10.3390/polym13081335

**Published:** 2021-04-19

**Authors:** Mohamed Ezzat Al-Atroush, Omar Shabbir, Bandar Almeshari, Mohamed Waly, Tamer A. Sebaey

**Affiliations:** 1 Department of Engineering Management, College of Engineering, Prince Sultan University, Riyadh 11543, Saudi Arabia; oahmed@psu.edu.sa (O.S.); balmeshari@psu.edu.sa (B.A.); tsebaey@psu.edu.sa (T.A.S.); 2 Department of Medical Equipment Technology, College of Applied Medical Sciences, Majmaah University, Majmaah 15341, Saudi Arabia; m.waly@mu.edu.sa; 3 Mechanical Design and Production Department, Faculty of Engineering, Zagazig University, Zagazig 44523, Egypt

**Keywords:** polyurethane foam, closed-cell, hydrophobic, expansive soil, stabilization, swelling potential, shrink–swell response

## Abstract

The reversible shrink–swell behavior of expansive soil imposes a serious challenge that threatens the overlying structures’ safety and durability. Traditional chemical additives such as lime and cement still exhibit satisfying performance over their counterparts in terms of swelling potential reduction. Nevertheless, significant concerns are associated with these chemicals, in addition to their environmental impact. This paper proposes a novel application of the closed-cell one-component hydrophobic polyurethane foam (HPUF) to stabilize the swelling soil. An extensive experimental study was performed to assess the efficiency of HPUF in mitigating both the swelling and shrinkage response of high montmorillonite content expansive soil. Expansive soil was injected/mixed with different weight ratios of the proposed stabilizer, and the optimum mixing design and injection percentage of the foam resin were identified to be ranged from 10% to 15%. The shrink–swell behaviors of both injected and noninjected samples were compared. Results of this comparison confirmed that HPUF could competently reduce both the swelling potential and the shrinkage cracking of the reactive expansive soil, even after several wet-shrink cycles.

## 1. Introduction

Several case studies available in the literature [1,2,3,4,5,6] reported the significant volume change of expansive soil, upon fluctuation in moisture content, as the principal reason behind the induced damages or cracking of the overlying structures or pavements. This problematic reactive soil is susceptible to shrinkage or swelling (heave) when its moisture content decreases or increases, respectively. With that in mind, lime and cement additives are the traditional efficient solutions that are commonly utilized to reduce the swelling potential (H_s_) associated with expansive soil. Despite the carbonation attacks, inefficiencies in controlling shrink–swell behavior of high sulfate expansive soils and their environmental have an impact on the greenhouse gas production salinization of water resources. 

Recently, new additives were adopted to treat the problematic swelling soil, such as the pozzolanic and recycled materials of fly ash, silica fume, etc. Several studies assessed the effectiveness of these new additives by comparing their effect with the effect of traditional solutions (lime or cement) in terms of the swelling pressure and potential. Among the different categories of additives employed for stabilizing expansive soils, the traditional chemical additives (lime and cement) still exhibit better performance over the other treatments, as concluded by Reddy et al. [7]. Despite this finding, new efficient alternatives should be proposed as stabilizers in order to overcome some of the problems associated with using lime in soil stabilization [8]. These stabilizers are not only expected to effectively reduce the volumetric strain associated with the expansive soil but also address the strength and the cost. In addition, the proposed stabilizers should also be environmentally friendly. Along the same line, several studies reported that polyurethane foam is mainly based on a three-dimensional, high-strength solid polymer produced with heat generation and increased volume when both liquid isocyanate and polyol (containing little water) compounds are combined. Compared with lime additive, both chemicals (isocyanate and polyol) are relatively environmentally friendly and long-lasting [9,10,11].

The current experimental study aspires to introduce a novel application for the closed-cell hydrophobic polyurethane foam (HPUF) in mitigating both the swelling and shrinkage response of the high montmorillonite content expansive soil. A comprehensive experimental study is performed to assess the efficiency of HPUF in stabilizing the swelling soil. Different weight ratios of the polymeric stabilizing agents are either injected into or mixed with the bentonite samples. The behavior of the expansive soil with and without the injected foam are compared to evaluate the capability of HPUF in reducing the swelling potential of expansive soil and to identify the optimum injection ratio.

## 2. Hypothesis

**Figure 1:**
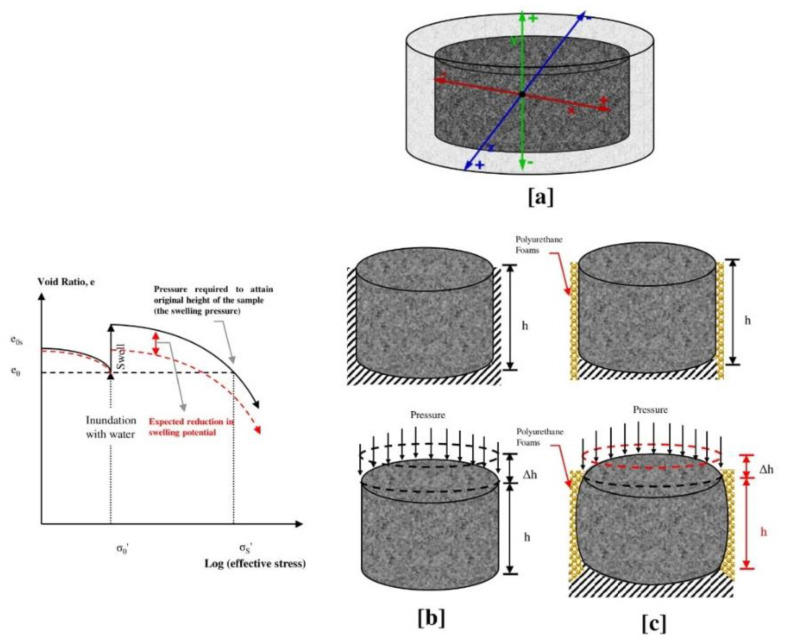
Expected swelling behavior of the expansive soil stabilized with the polyurethane foam material (adapted from Al-Atroush and Sebaey [8]). (**a**) Not-restrained sample (**b**) Restrained sample in lateral direction. (**c**) Stabilized and restrained sample.

Many studies discussed the swell–shrink mechanism of expansive soil [12,13,14,15,16]. It was concluded that the expansive soil may swell freely in the three dimensional with equal magnitudes along the principal axes (Figure 1a), in case the expansive soil is not restrained (i.e., free in three-dimensional form). However, in real engineering practice, the soil is frequently restrained in the lateral direction. Therefore, in most cases, the swelling action is predominantly expected to occur in the vertical direction predominantly (Figure 1b). On the other side, with the decrease in moisture content (which can occur due to evaporation process, for instance), the tensile stresses and strains in the soil begin to be developed due to the influence of surface tension (suction), which causes shrinkage cracks in the expansive soil. This shrinking action causes settlement at the ground surface due to the soil volume decreasing in both the vertical direction and the soil fracture that is expected to occur due to the soil volume decreasing in the lateral direction [17].

With that in mind, the injection of energy-absorbing material such as polyurethane foam into the swelling soil is expected to compress it horizontally and/or vertically. Consequently, this reduces the vertical soil heave by absorbing a large amount of the soil energy once the PU foam is compressed in the horizontal direction, as shown in Figure 1c. In addition, the polymer agent may superglue the soil particles together and produce a more stabilized system that can resist the volume change. 

## 3. Materials and Engineering Properties

The mineralogical, physical, and chemical properties of the selected high montmorillonite content expansive soil and the mechanical properties of the hydrophobic closed-cell polyurethane foam (HPUF) are also identified and discussed in this section.

### 3.1. Expansive Soil Material 

**Figure 2:**
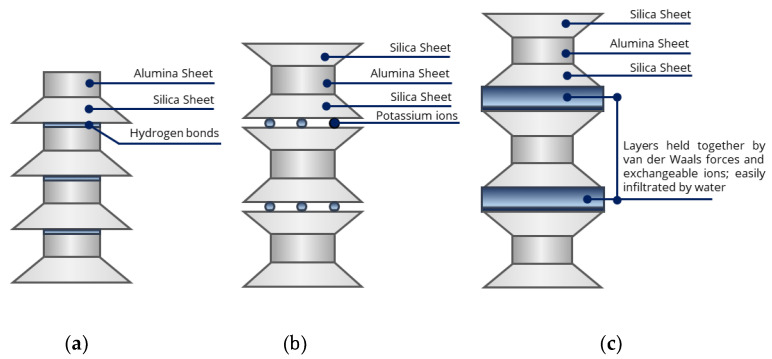
Main groups of clay minerals. (**a**) Kaolinite (1:1 non-expanding); (**b**) illite (2:1 non-expanding); (**c**) montmorillonite (2:1 expanding) [18].

The degree of expansion depends upon the type and amount of clay minerals and exchangeable ions [18]. Two principal units, i.e., alumina octahedron and silica tetrahedron, compose the clay minerals’ aluminum silicates (Figure 2). The main groups of clay minerals are illite, kaolinite, and montmorillonite. Kaolinite and illite are mostly inert and are proof against water penetration because of the strong and stable bonding between their layers in the existence of water. In contrast, the bonds between montmorillonite layers are weak and easy to separate in the presence of water, thus causing the soil to expand due to the increase in water intrusion inside the soil mass, as shown in Figure 2. Existing montmorillonite in the soil creates most of the expansive soil problems [18,19]. The increase of montmorillonite content in the expansive clays increases both the swelling potential and pressures [16].

Bentonites are high-swelling clays with low hydraulic conductivity (k) that are commonly used in a wide range of geotechnical and geoenvironmental applications. With that in mind, because of its high montmorillonite content, the pure high swelling sodium-based bentonite (Na-bentonite) provided by Nano Technologies Co. (Alexandria, Egypt) was specially selected for this experimental study. 

The product manufacturer provided the specific gravity and dry density, and some other Na-bentonite index properties were obtained from different soil laboratory tests that were performed through this study. The physical and chemical properties and the mineralogical composition of the Na-bentonite are summarized in Table 1. This Na-bentonite contained 92% clay-sized particles [20] and is classified as high-plasticity clay (CH) in accordance with the Unified Soil Classification System [21]. The bentonite’s mineralogical composition (X-ray diffraction) was 91% montmorillonite, 3% plagioclase feldspar, 2% quartz, 2% illite/mica, and 2% other minerals, as reported by the product manufacturer. Consistency of liquid and plastic limits were obtained as 428% and 34%, respectively, in accordance with ASTM D4318-10 [22]. 

### 3.2. Polyurethane Foam Materials

**Figure 3:**
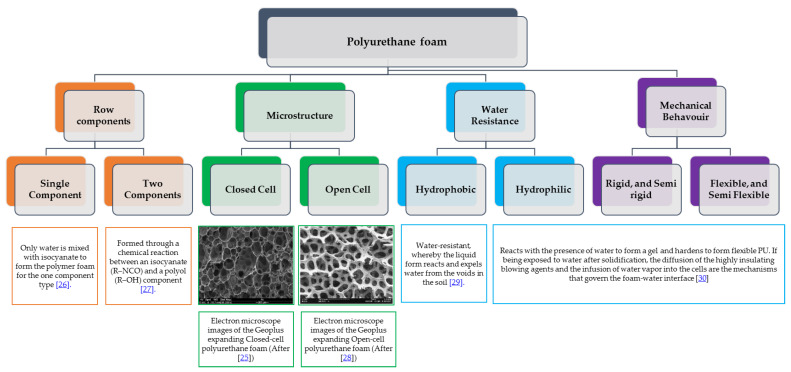
Types and main characteristics of polyurethane foam materials.

Polyurethane foam materials are classified as polymer materials. They are commonly used as protection from impacts and to absorb energy [24]. Polyurethane (PU) foam can be found in various forms and can cover a wide variety of properties and, therefore, applications. They are commonly used in many applications such as packaging, cushioning, space-filling, and insulation. PU foam resin is a fluid substance that can be quickly injected into the soil. Nevertheless, polyurethane (PU) was rarely used in geotechnical engineering applications [25]. Figure 3 summarizes the different types of PU foam, and the main characteristics for each type, according to the row components required for chemical interaction, microstructure, water resistance, and its mechanical behavior [25,26,27,28,29,30]. More details about each type can be found in [8]. For the proposed application, closed-cell one-component hydrophobic polyurethane foam (HPUF) is adopted in the current study. The provided properties for this material by the manufacturer are given in Table 2.

The use of expanding polyurethane as a filling and lifting agent in soft soils was reported as an effective stabilizer in many cases, specifically for treating problems associated with excessive and differential settlements. The high expansion characteristics of the polyurethane foam volume were beneficial in maintaining deformed pavement, buildings, and other underground construction, where traditional or conventional techniques were infeasible [31,32]. Limited experimental studies pinpointed several positive signs for using polyurethane foam agents as a soil stabilizer [8]. 

## 4. Methodology

An extensive experimental study was performed to assess the efficiency of the closed-cell hydrophobic polyurethane foam (HPUF) in mitigating both the swelling and shrinkage response of the high montmorillonite content Na-bentonite clay. For this purpose, the experimental study included three main parts. First, the swelling–shrinkage behavior of the Na-Bentonite clay was examined. The swelling potential (H_s_), shrinkage cracking, and swelling soil response after several wet-shrink cycles were determined utilizing the Oedometer test [33], water loss shrinkage test, and free swell test, respectively. 

Since the energy absorption capacity of HPUF is affected by its density, it was fundamental to perform several mixes and investigate the effect of each mixing component percentage on the resulted density. With that in mind, in the second part of the experimental study, the relation between the foam density and the mechanical properties was obtained by testing the prepared foam samples in compression according to ASTM D1621-16 [34]. The energy absorption capacity was the main governing factor used for adopting the optimal mixture composition, as it leads to the optimal energy absorption capacity.

The third part of the experimental study was mainly concerned with assessing the efficiency of HPUF in swelling reduction. This was explored by injecting/mixing the adopted HPUF optimum mixture composition into the expansive soil sample with different weight percentages of 1%, 5%, 10%, and 15%. The behavior of the injected/mixed soil samples was compared with the behavior of noninjected/mixed samples in terms of swelling potential, expansion ratio with time, shrinkage cracking, and swelling response after three complete wet-shrink cycles. 

### 4.1. Na-Bentonite Specimens Preparation and Testing

**Figure 4:**
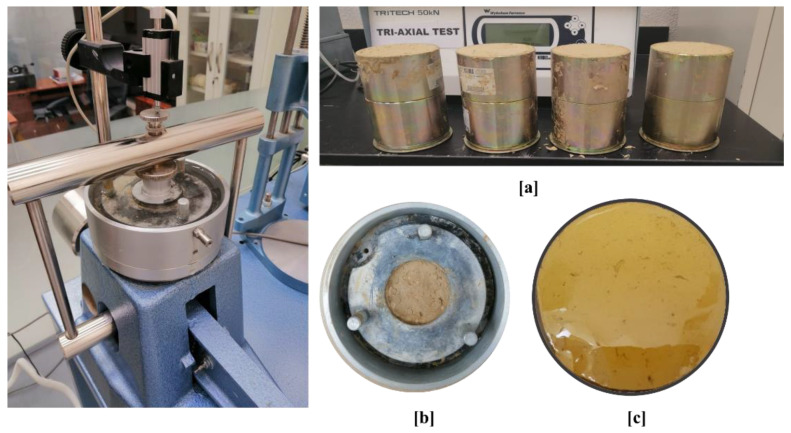
Na-bentonite specimens preparation and testing: (**a**) samples prepared for compaction test; (**b**) samples prepared for oedometer test; (**c**) samples prepared for shrinkage test.

Compaction tests using the standard Proctor compaction method [35] were carried out to obtain the optimum water content and maximum dry unit weights of the Na-bentonite specimens. Samples for five mixtures were prepared with water contents ranging from 5% to 25% (Figure 4a). Distilled water was added to the dry bentonite powder. The dry unit weight and water content of the compacted Na-bentonite mixtures were determined, and the compaction curve was plotted. Thus, the maximum dry unit weight and the optimum water content of the selected Na-bentonite compacted mixture that is utilized in this study were identified. 

One-dimensional swelling tests were carried out following ASTM D4546-96 [36] (method A). The fixed ring apparatus is an oedometer in which the ring holding the sample was not allowed to move during testing (Figure 4b). A chamber surrounding the ring was used for the submergence of the sample. Two porous disks with filter papers next to the sample were placed at the bottom and the top of the sample. The oedometer cell was placed within a loading frame, which transmitted vertical loads through the lever arm. A dial gauge of an accuracy of 0.01 mm was used to monitor the sample height change. Digital dial gauges connected to a data logger were used in some of the experiments. Front-loading frames were used to apply a weight of 6.9 kPa to the sample. The diameter and the height of the ring used were 50 mm and 20 mm, respectively. Two samples were submerged and were allowed to swell vertically at the seating pressure until the primary swell was complete. The oedometer tests were performed on two samples of mixtures that were prepared using dry bentonite powder mixed with the optimum water content obtained from the compaction test. The two samples prepared were placed in the oedometer ring and leveled off prior to placement in the oedometer cell. The weight of the sample was determined as 58.5 g. The initial height of the sample was recorded. The initial moisture content and specific gravity were obtained. These measures enabled the swelling potential (H_s_), to be computed with time.

On the other side, the induced cracks due to the expansive soil’s shrinkage were explored. Bentonite samples were prepared with a similar approach and placed in 100 mm plastic transparent plates (Figure 4). The loss in specimen volume and the cracking propagation were monitored with time for 120 h.

**Figure 5:**
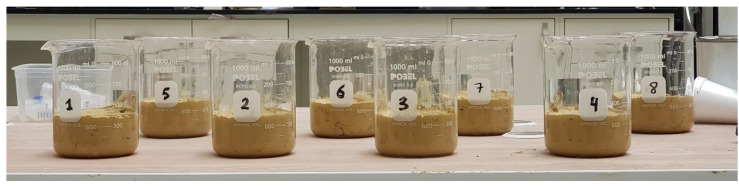
Large-scale free swell arrangement prepared to test Na-bentonite specimens in wet-shrink cycles.

In addition, eight bentonite samples with a 100 mm diameter and 300 mL volume were prepared and tested in three wet-shrink cycles through a large-scale free swell arrangement to assess the change in swelling behavior after different cycles (Figure 5). In each cycle, the bentonite specimens were submerged in 900 mL of distilled water, and the swelling potential results were taken after 72 h. Around 21 days was the time required for water evaporation, and the samples were allowed to shrink for another five days, which means that each cycle takes around one month (30 days).

### 4.2. Characterization of Hydrophobic Polyurethane Foam (HPUF) Samples

In this study, the one-component, closed-cell hydrophobic polyurethane foam (HPUF) was utilized. For this PU foam class, water is the only blowing agent that affects the formation of the hardened foam; 10% is the recommended catalyst ratio to be added to the polymer resin in order to optimize the chemical reaction time. However, no recommendation was provided by the manufacturer for the optimal water content. Therefore, four different mixes were conducted to investigate the effect of exothermic reaction between the foam resin and the water content on the microstructure, density, and mechanical properties of the produced semirigid PU foam material. Water contents adopted for these four mixes were 10%, 20%, 50%, and 100%. In addition, 30 mL and 3 mL were the volumes utilized for the foam resin and catalyst, respectively, in the four mixes. One fundamental point to note is that all mixes were prepared at the same temperature (20 °C). The foam expansion ratio, hardening time, and density were obtained for the four mixes with different water contents. This experiment was repeated three times, and the error percentages were determined.

**Figure 6:**
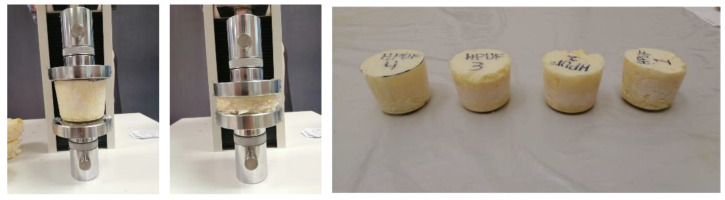
Prepared samples of closed-cell hydrophobic polyurethane foam (HPUF) with various densities and the compression test setup.

To investigate the effect of the foam density on the mechanical behavior of the HPUF, four samples were prepared with the same percentages addressed above to be tested under compression stress. The four samples were cast in a custom cylindrical stainless-steel mold with an extruder. According to ASTM D1621-16 [34], sharp cutting tools were used to prepare the four samples with a 500 mm diameter and a height of 510 mm to maintain the ratio between the height and the diameter and to avoid any buckling effect during the compression test (Figure 6). Each sample’s compression stress was obtained at both 10% and 90% strain percentages; these values represent the foam material’s mechanical behavior at both elastic and failure stages [37]. In addition, each foam sample’s strength and energy absorption capacity were determined using the method explained in ASTM D1621-16 [34].

### 4.3. Preparation of the Injected/Mixed Bentonite Samples with HPUF

Na-bentonite samples were prepared with the similar approach explained in Section 4.1. Injection and mixing stabilizing techniques were used to insert the HPUF liquid resin into the bentonite samples. Four bentonite samples were injected using a fine injection tool (air pressure). Before the complete chemical action (i.e., before hardening), the HPUF was injected. The HPUF was injected in a triangular pattern to maintain the uniformity of the mixture. The four samples were injected with 1%, 5%, 10%, and 15% HPUF weight ratios to be examined in the one-dimensional oedometer test swelling test [33]. In addition, another four bentonite samples were mixed with 1%, 5%, 10%, and 15% HPUF weight ratios to be examined in the shrinkage apparatus, and a large-scale free swell test set up was prepared to monitor the response of samples after three full wet-shrink cycles. The two experiments were reperformed four times, and the error percentages were determined.

## 5. Results and Discussion

This section discusses the efficiency of the HPUF in mitigating both the swelling and shrinkage response of the high montmorillonite content expansive soil. The HPUF’s efficiency was assessed by comparing the behavior of the injected/mixing soil samples with the original Na-bentonite samples’ behavior in terms of the swelling potential, expansion rate with time, induced shrinkage cracking, and swelling response after three full wet-shrink cycles.

### 5.1. Swell–Shrink Behavior of Na-Bentonite

**Figure 7:**
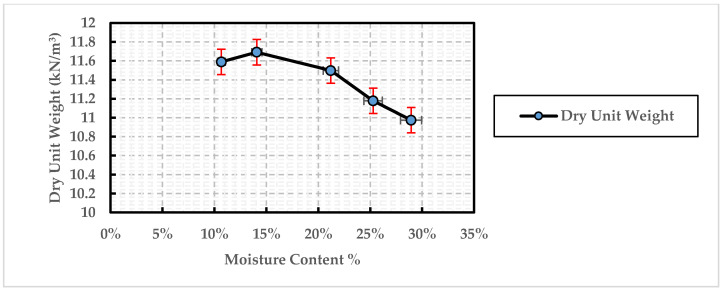
Results of compaction test performed on Na-bentonite samples.

The maximum dry unit weight and the optimal moisture content of the Na-bentonite were determined through the compaction test. As shown in Figure 7, the maximum dry unit weight was 11.7 kN/m^3^ when the optimal water content was considered as 15%. One fundamental point to note is that the obtained optimal moisture content was adopted for preparing all bentonite samples examined within this study.

**Figure 8:**
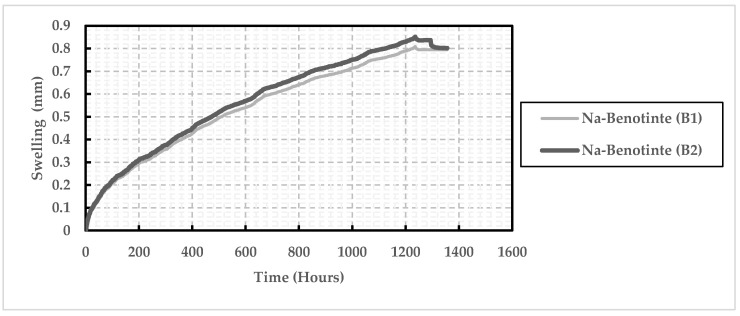
Results of one-dimensional swelling test carried out for two original bentonite specimens.

Results of the one-dimensional swelling test performed on the two Na-bentonite samples indicated that bentonite samples started to swell and increase in volume once they were submerged. As shown in Figure 8, the swelling potential increased to reach it maximum value of 0.837 mm after about 1180 h. This behavior was almost consistent for the two samples examined in the oedometer test. After 1354 h, the swelling potential tends to be constant with a value equal to 0.802 mm, representing a 104% swelling ratio.

**Figure 9:**
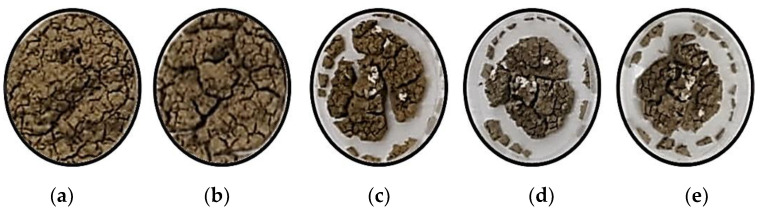
The induced cracks due to the shrinkage of the expansive soil. (**a**) After 24 h. (**b**) 48 h. (**c**) 72 h. (**d**) 96 h. (**e**) 120 h.

On the other side, the loss in specimen volume and the cracking propagation were monitored for 120 h. Figure 9 shows the changes in mass loss and the cracking propagation of the bentonite specimen with time change. The evaporation of water is the main reason for the mass change. With a large amount of water evaporated, a significant mass loss of about 24% was observed in the bentonite samples.

**Figure 10:**
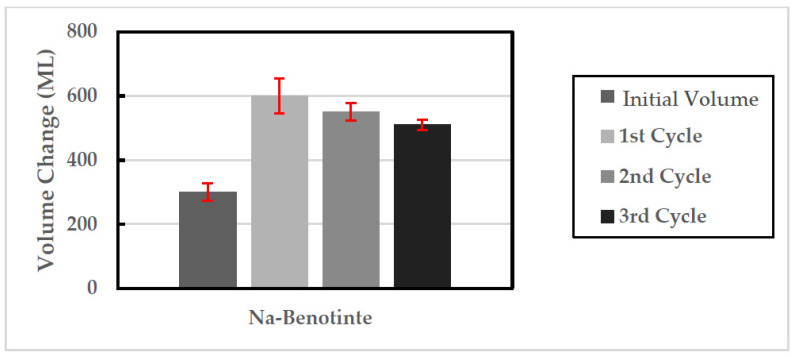
Comparison between the obtained average swelling potential after three full wet-shrink cycles (three months).

Figure 10 compares the two original bentonite samples’ obtained average free swelling potential before and after submergence in the water, along with three full wet-shrink cycles (three months). As shown, swelling percentages of 200%, 183.3%, and 170% were obtained in the 1st, 2nd, and 3rd cycles, respectively. 

### 5.2. Mechanical Behavior of the Hydrophobic Polyurethane Foam (HPUF)

The first expectation for using the PU foam as a stabilizer for expansive soil was that it would act as a compressible material with high energy absorption capacity and reduce the soil’s swelling potential. Existence of this compressible media may reduce the restraints in the lateral direction to decrease the expected swelling in both vertical and lateral directions. This concept is close to expanded polystyrene (EPS) geofoam’s mechanism discussed by Ikizler et al. [38]. However, HPUF may be highly practical in the field as it can be injected as a liquid and can harden inside the soil voids in a very short time. Results of the foam densities and the hardening time obtained for the four mixes with different water content are given in Table 3. These results confirmed that the hardening time of the HPUF ranges from 9 min to 90 min upon the water content utilized in the mix. 

**Figure 11:**
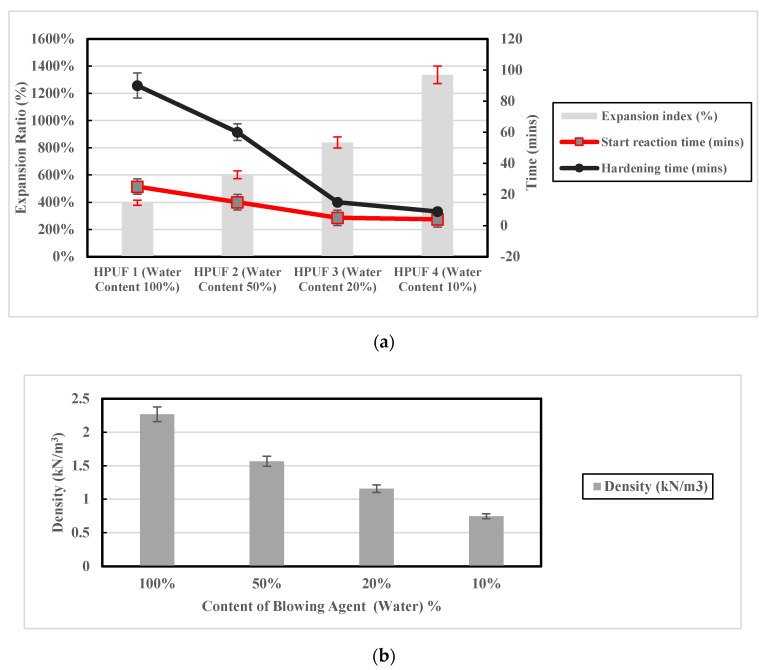
Comparison between the characteristics of four different mix designs of HPUF with various water contents: (**a**) expansion ratio and reaction time; (**b**) density.

It also can be seen from Figure 11a that the increase in the content of the blowing agent (water) causes an increase in the chemical reaction start time and the time required for the full exothermic reaction (hardening state). The first sample with 100% water content takes about 90 min for the full reaction and forms solid foam. However, this time is reduced to be only 9 min when the water content decreased to 10% (i.e., the fourth mix). Conversely, the increase in water content decreases the hardened foam’s expanded volume (expansion ratio). 

Figure 11b shows the influence in foam density (at the solid state) with the water content change. It was clearly observed that the densities of the HPUFs increase with the increase in water content. It was also noted that the first sample (100% water content) was relatively dense and contained some traces of water, which means that some of the water used in the mix did not interact in the chemical action. In contrast, the fourth sample was very flexible, and no water trace was noted after the hardening. 

**Figure 12:**
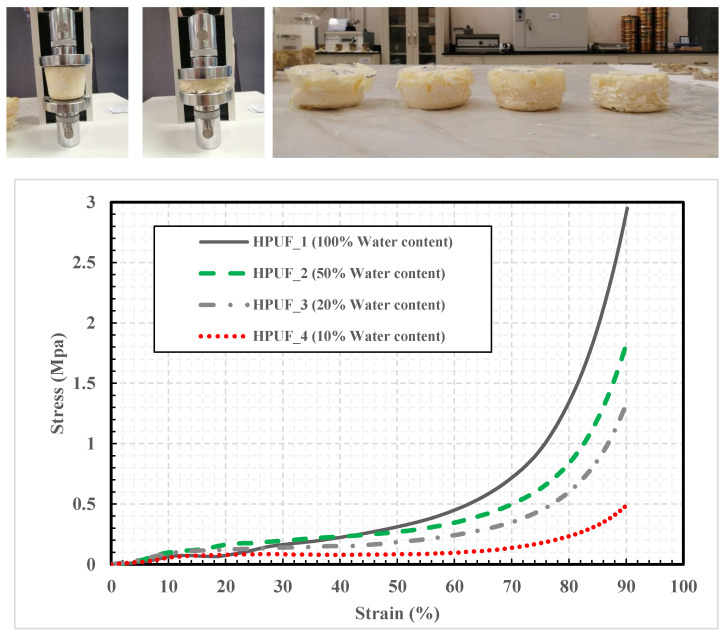
Results of the compression test performed for the four foam samples with different densities.

The four HPUF samples were also tested under compression stress according to ASTM D1621-16 [34]. Table 4 summarizes the four foam samples’ mechanical properties with different densities (water contents), including the strength and energy absorption capacity. In addition, Figure 12 shows the stress–strain curves of the four HPUF samples with different densities at both 10% and 90% strain percentages.

It can be seen from Table 4 that the density increases from 0.517 to 1.566 kN/m^3^, and the compressive stress at 10% strain of the PUFs increases from 0.055 to 0.098 MPa. However, the result of the first foam sample was inconsistent with those of the other three. Despite the high density of the first sample (HPUF 1), it required less compressive stress to achieve the 10% strain level than the second foam sample (HPUF 2). This can be explained by the lower expansion ratio of the first foam sample caused by the formation of relatively rigid walls around the foam cell. 

Toughness is the material’s resistance to fracture or ability to undergo deformation, and it represents the energy absorption capacity. It can be calculated from the area under the curve in the stress–strain curve. Figure 12 shows the effect of density on the toughness or energy absorption of the PUF. The results show that toughness or energy absorption increases with the increase in density. 

**Figure 13:**
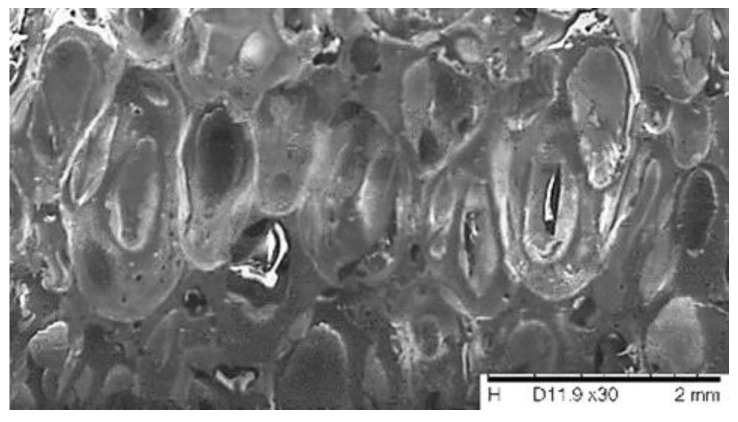
Electron microscope images of the hydrophobic polyurethane foam (HPUF 2).

Table 4 shows that the second mix (HPUF 2) is superior to the other three in energy absorption capacity, strength, and compressibility at both the elastic and failure phases. Therefore, the percentage of the component utilized in preparing the second foam mix, i.e., HPUF 2 (50% water content), was considered as the optimal content and was consequently adopted to be used in the soil stabilization tests. The microstructure of this foam (HPUF 2) was explored using an electronic microscope (SEM), as shown in Figure 13.

### 5.3. Efficiency of the HPUF in Reduction of Soil Swelling Potential

**Figure 14:**
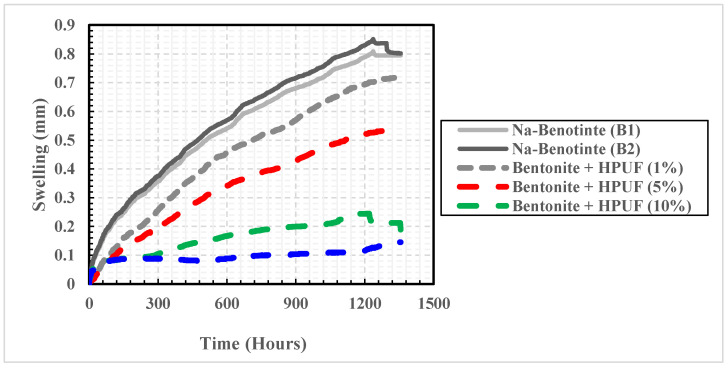
Results of one-dimensional swelling test carried out for two noninjected bentonite specimens and four injected ones with different foam weight ratios.

To explore the effect of HPUF on swelling behavior and obtain the optimal injection percentage, a series of swelling tests under constant stress (6.9 kPa) were conducted using an oedometer apparatus [33]. Results of the four injected samples with different foam weight ratios (1%, 5%, 10%, and 15%) were compared with the two original noninjected samples. The six samples were allowed to swell for up to 2 months. The results of the one-dimensional swelling tests are shown in Figure 14.

Figure 14 indicates that the response of the noninjected specimens (B1 and B2) was fairly consistent. In contrast, the swelling behavior of the injected specimens varied significantly in both magnitude and rate. The injected specimens consistently swelled much less than the noninjected specimens. However, the reduction rate did not linearly increase as the foam percentage increased. In particular, the injected specimens with HPUF with weight ratios of 1%, 5%, 10%, and 15% have less swelling with percentages of 16%, 36.6%, 74.16%, 81.84%, respectively, compared to the noninjected samples. It was also observed that the reduction in swelling potential became almost halved when the HPUF percentage increased from 1% to 5% and from 5% to 10%. However, only a 7% extra decrease in the swelling potential was noted when the HPUF percentage increased from 10% to 15%. Thus, a 10% injection percentage can be described as optimal.

### 5.4. Efficiency of the HPUF in Eliminating the Shrinkage Cracking

**Figure 15:**
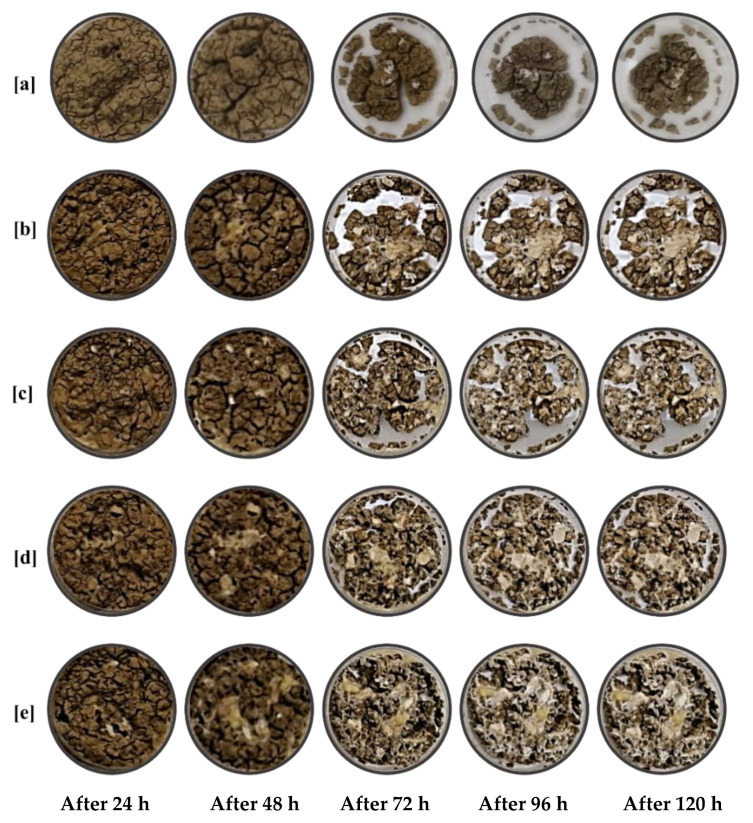
Effect of HPUF on the reduction of the induced cracks due to the shrinkage of the expansive soil: (**a**) Injected 0%HPUF; (**b**) injected 1% HPUF; (**c**) 5% HPUF; (**d**) 10% HPUF; (**e**) 15% HPUF.

The effect of HPUF on reducing the induced cracks due to the shrinkage of the expansive soil was explored through four injected Na-bentonite specimens with 1%, 5%, 10%, and 15% HPUF weight ratios. The loss in specimen volume and the cracking propagation were monitored for 120 h, as presented in Figure 15.

Figure 15 highlighted that with the large amount of water evaporated at the initial time, a significant mass loss was observed in all samples, which ranged from 18% to 24%. With time, less water amount was retained in the specimens, leading to a slow decrease in the mass-loss rate. When the PU foam was added at a relatively low weight ratio (1% and 5%), bentonite specimens presented almost a similar behavior. At the beginning of evaporation, HPUF could improve the cracking properties by stabilizing more water into the bentonite specimens. The mass of the injected samples with 10% and 15% (Figure 15d,e) decreased less compared with the noninjected specimen (Figure 15a). The existence of the HPUF helps in stabilizing the retained water for a longer time, and therefore water evaporated more gradually from the samples, which reduced the shrinkage cracks in the last two specimens. 

### 5.5. Behavior of the Expansive Soil Mixed with HPUF in Wet-Shrink Cycles

**Figure 16:**
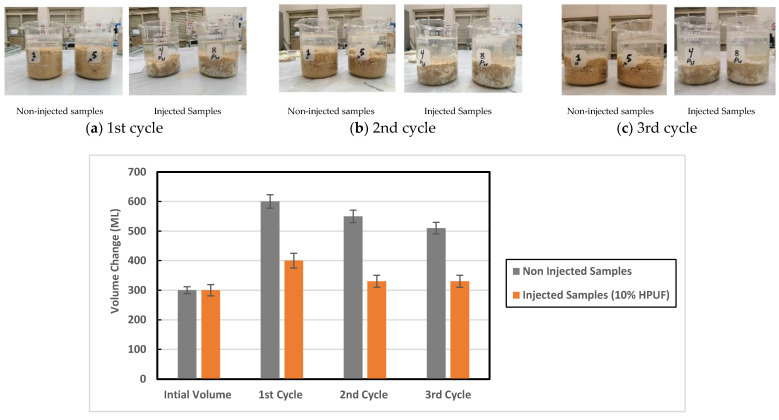
Comparison between the obtained average swelling potential of two noninjected and two injected samples after three full wet-shrink cycles (three months).

The efficiency of the HPUF in mitigating the swelling potential of the expansive soil after several wet-shrink cycles was assessed through a large-scale free swell arrangement that was used to test four bentonite samples. The first two bentonite specimens were noninjected (original), and the other two were mixed with 10% of the HPUF. In each cycle, the four bentonite specimens were submerged in 900 mL of distilled water, and swelling potential results were taken after 72 h. Around 21 days was the time required for water evaporation, and the samples were allowed to shrink for another 5 days. Figure 16 compares the obtained average swelling potential of the original samples with the average one obtained for the two samples mixed with 10% HPUF, along with three full wet-shrink cycles (three months).

As shown in Figure 16, the mixing of HPUF with a weight ratio of 10% succeeded in reducing the swelling potential of the expansive soil in the three wet-shrink cycles, as these samples showed less swelling than the original samples with differences of 50%, 67%, 54% in the first, second, and third cycles, respectively.

According to the experimental study [7], lime treatment (i.e., the traditional chemical treatment solution) was superior to other nontraditional additives such as fly ash, stone dust, and silica foam. Using lime as a treatment additive succeeded in reducing the expansive soil’s swelling potential with a percentage of about 60%. By comparing this percentage with the one obtained in this study using the HPUF, it can be concluded that using HPUF may be a promising treatment solution for expansive soil and is able to reduce the soil swelling potential with a comparable percentage to the lime. 

As a future research line, the paper reveals an approach that can be reutilized to evaluate the efficiency of the HPUF in mitigating the swelling stress of expansive soil. This will be beneficial to monitor and study the response of expansive soil with the HPUF stabilizer. In addition, experimental studies may extend this research by investigating the effect of the HPUF on soil bearing capacity and other soil’s compressibility properties.

Field studies should be carried out to investigate the different injection and mixing methods and highlight the superior implementation technique that fits HPUF characteristics and leads to the maximum reduction in soil swelling. Moreover, the field study may also investigate the effective performance of the HPUF over time and under different destructive chemical, physical, and mechanical conditions. This can be done through different aging tests.

Similar to EPS foam, the HPUF can be implemented as inclusions. However, numerical analyses should be performed to explore factors affecting the stabilized soil’s swell–shrink mechanics using this suggested stabilizer. This parametric study may include the effect of inclusion diameter and length, the spacing between the foam columns, and the effect of the permeability parameters for both the soil and foam material.

## 6. Conclusions

This paper introduced a novel application of the closed-cell, one-component hydrophobic polyurethane foam (HPUF) to be used as a swelling soil stabilizer. Results of the laboratory tests performed confirmed that HPUF efficiently succeeded in mitigating both the swelling potential and the shrinkage cracking of the reactive expansive soil, even after several wet-shrink cycles. In addition, based on the results of the experimental study performed, the following points can be drawn:

- HPUF could competently reduce both the swelling potential and the shrinkage cracking of the reactive expansive soil, even after several wet-shrink cycles.
- Single-component polyurethane foam’s mechanical properties, including density, reaction time, the expansion ratio, are affected by the water content (blowing agent) that is added for the chemical reaction during the formation process.
- The density of the HPUF increased with the increase in water content. The mechanical properties of the foam sample with 50% water content were superior to the other three samples in terms of energy absorption capacity, strength, and compressibility at both elastic and failure phases.
- Results of free swell tests showed that HPUF could successfully reduce the swelling potential of the expansive soil with an average reduction percentage of about 50%, and the optimal injection percentage can be ranged from 10% to 15%.
- The analyses involving the cracking patterns and the change of moisture content with time showed that HPUF significantly contributes to stabilizing the retained water for a longer time into the soil. Therefore, water evaporated more gradually from the samples, which pointedly reduced the shrinkage cracks in the specimens injected with relatively high weight ratios (10% and 15%).
- In particular, the HPUF stabilizer was able to mitigate the expansive soil’s swelling potential along three different wet-shrink cycles. This may be attributed to the polymer’s ability to generate a nanocomposite structure that gives the soil more resistance against the volume change, in addition to its ability to superglue the soil particles and produce a more stabilized system that can resist the volume change.
- Dissimilar to lime and fly ash, HPUF does not require curing, thus requiring shorter construction times. In addition, HPUF resin can be mixed or injected into the soil. These key merits empower the HPUF to be a comparable stabilizer that can be used to overcome the associated problems with the traditional chemical stabilization methods.

## Figures and Tables

**Table 1:** The mineralogical, physical, and chemical properties of Na-bentonite.

Property	Average Value or Type (Number of Trials Performed)	Reference or Standard
**Specific gravity**	2.71 (10)	[23]
**Density**		Product manufacturer (data sheet)
Bulk density	917 kg/m^3^	
Dry density	860 kg/m^3^	
**Soil classification (USCS)**		
Sieve analysis (air-dried)	SP (3)	[21]
Hydrometer	CH (3)	[21]
**Clay (%)**	96% through 125-micron (μm) sieve	[20]
**Water content**	11%	Product manufacturer
**Atterberg limits (%)**		[22]
Liquid limit, LL	428 (3)	
Plastic Limit, PL	34 (3)	
Plasticity index, PI	394 (3)	
**Saturated soil paste**		Product manufacturer (data sheet)
pH	8.9	
Electrical conductivity	305	
**Principal minerals (%)**		Based on X-ray diffraction (XRD) analyses performed by the product manufacturer
Montmorillonite	91	
Quartz	2	
Plagioclase feldspar	3	
Calcite	1	
Ferroan dolomite	Trace	
Gypsum	1	
Illite/mica	2	

**Table 2:** Properties of HPU foam.

	Isocyanate	Catalyst	Cured Foam
**Viscosity at 25 °C (mPas)**	Approx. 350	Approx. 15	
**Density (kg/dm^3^)**	Approx. 1.075	Approx. 0.950	
**Compressive Strength (MPa)**		3 (10%)	Approx. 9.5
**Flexural Strength (MPa)**	30	3 (10%)	Approx. 9

Note: Source of data is the product manufacturer data sheet.

**Table 3:** Four different mix designs of PU foam resins.

Sample	Foam Resin	Catalyst	Distilled Water	Water Content	Expansion Ratio V_Initial_/V_Final_	Density	Start Reaction Time	Hardening Time
Units	(mL)	(mL)	(mL)	(%)	(%)	(kN/m^3^)	(min)	(min)
**HPUF (1)**	30	3 (10%)	30	100%	396.86%	2.267	25	90
**HPUF (2)**	30	3 (10%)	15	50%	601.02%	1.566	15	45
**HPUF (3)**	30	3 (10%)	6	20%	838.34%	1.094	5	13
**HPUF (4)**	30	3 (10%)	3	10%	1335.59%	0.517	4	9

**Table 4:** Mechanical properties of the four HPU foam samples.

	Density (kN/m^3^)	Loading Rate (mm/min)	Compressive Stress at 10% Strain (MPa)	Compressive Stress at 90% Strain (MPa)	Strength (MPa)	Absorbed Energy (J)
**HPUF (1)**	2.267	2.50	0.063	2.87	69.25	58.52
**HPUF (2)**	1.566		0.098	1.707	104.67	42.84
**HPUF (3)**	1.094		0.092	1.31	99.46	30.73
**HPUF (4)**	0.517		0.055	0.46	62.08	13.23

## Data Availability

Not applicable.
